# Unilateral Cleavage Furrows in Multinucleate Cells

**DOI:** 10.3390/cells9061493

**Published:** 2020-06-18

**Authors:** Julia Bindl, Eszter Sarolta Molnar, Mary Ecke, Jana Prassler, Annette Müller-Taubenberger, Günther Gerisch

**Affiliations:** 1 Max Planck Institute of Biochemistry, Am Klopferspitz 18, D-82152 Martinsried, Germany; j.bindl@campus.lmu.de (J.B.); eszter.molnar@campus.lmu.de (E.S.M.); ecke@biochem.mpg.de (M.E.); prassler@biochem.mpg.de (J.P.); 2 LMU Munich, Department of Cell Biology (Anatomy III), Biomedical Center, D-82152 Planegg-Martinsried, Germany; amueller@bmc.med.lmu.de

**Keywords:** cell fusion, cortexillin, cytokinesis, *Dictyostelium*, myosin

## Abstract

Multinucleate cells can be produced in *Dictyostelium* by electric pulse-induced fusion. In these cells, unilateral cleavage furrows are formed at spaces between areas that are controlled by aster microtubules. A peculiarity of unilateral cleavage furrows is their propensity to join laterally with other furrows into rings to form constrictions. This means cytokinesis is biphasic in multinucleate cells, the final abscission of daughter cells being independent of the initial direction of furrow progression. Myosin-II and the actin filament cross-linking protein cortexillin accumulate in unilateral furrows, as they do in the normal cleavage furrows of mononucleate cells. In a myosin-II-null background, multinucleate or mononucleate cells were produced by cultivation either in suspension or on an adhesive substrate. Myosin-II is not essential for cytokinesis either in mononucleate or in multinucleate cells but stabilizes and confines the position of the cleavage furrows. In fused wild-type cells, unilateral furrows ingress with an average velocity of 1.7 µm × min^−1^, with no appreciable decrease of velocity in the course of ingression. In multinucleate myosin-II-null cells, some of the furrows stop growing, thus leaving space for the extensive broadening of the few remaining furrows.

## 1. Introduction

Mitotic cell division is typically mediated by a contractile ring that, after segregation of the chromosomes, forms a cleavage furrow to separate the daughter cells. Double-headed myosin II has been shown to be a common driver of ring constriction in cells as divergent as *Schizosaccharomyces pombe*, sea urchin blastomeres, and mammalian cells [1,2]. Constriction of the ring is based on the interaction of the bipolar filaments of myosin-II with anti-parallel actin filaments that are linked to the membrane [3]. 

Nevertheless, there are exceptional modes of cleavage. First, in a variety of parasitic protozoans, cytokinesis is performed without the participation of myosin-II [4]. Second, mitotic cleavages can be accomplished by furrows that ingress laterally from one side of the cleavage region. The formation of unilateral furrows raises two principal questions. The first one is: how do these furrows progress to separate the daughter cells? The second question is: can mitotic cells alternate between cytokinesis by normal, ring-shaped cleavage furrows and by unilateral ones? This would suggest that the machinery responsible for cytokinesis is flexible enough to perform the constriction of a cytokinetic ring as well as the unilateral ingression of a furrow. Here, we used mono- and multinucleate cells of the eukaryotic microorganism *Dictyostelium discoideum* to address these questions. 

Unilateral furrows are of general interest since they are formed under various conditions in cells other than the multinucleate *Dictyostelium* cells. In *Physarum polycephalum*, unilateral furrowing occurs during transition from the mononucleate amoebal state to the multinucleate plasmodium [5]. This transition is accomplished by the switch from astral mitosis connected with cytokinetic furrowing to anastral mitosis, with an intranuclear spindle that omits cytokinesis. The formation of incomplete cleavage furrows as intermediates between complete cytokinesis and lack of it is favored by the presence of additional microtubule-organizing centers, which unilaterally prevent the formation of a furrow.

Similarly, intracellular particles can asymmetrically block the formation of a cleavage furrow in HeLa cells infected with *Chlamydia*; the bacteria causing these human cells to form a unilateral furrow. At the side close to the inclusion of *Chlamydia* particles, the accumulation of RhoA became attenuated [6], and consequently the accumulation of anillin and the assembly of myosin-II were inhibited. The asymmetric accumulation of RhoA was due to the truncated localization of a RhoGEF, Ect2. In line with these observations, a unilateral furrow could be experimentally induced in HeLa cells using optogenetics to locally activate Ect2 in one area of the cleavage plane [7].

Unilateral furrows with microfilaments decorating the cleavage zone in an “arcuate manner” are formed in cnidarian eggs of the genus *Aequorea* [8]. These furrows begin at the animal pole and ingress toward the vegetal pole. In amphibian embryos, isolated blastomeres of the outermost layer, called “superficial cells”, form unilateral furrows beginning at their adhesive basal surface and progressing toward their non-adhesive apical surface [9]. A unilateral furrow is also formed at the basal region of *Echinarachnius* (sand dollar) eggs that are forced into a conical shape [10]. 

Related to unilateral furrowing is the ingression of furrows between nuclei during blastoderm formation in early insect embryogenesis [11]. The subsequent constriction of a ring at the blastoderm–yolk interface occurs in two steps: a first slow phase and a second faster phase. Only the first phase is myosin-II-dependent [12].

During meiosis II in mouse oocytes, a unilateral furrow initiates polar body formation [13]. After turning of the spindle, this furrow is converted into a bilateral one or a contractile ring (reviewed by Uraji et al. [14]). Finally, there are unilateral cleavage furrows formed in electrofused mammalian cells, as reported for PtK1 cells by Savoian et al. [15]. 

In summary, there are three conditions under which unilateral cleavage furrows have been observed: (1) during plasmodium formation as an intermediate state between mitosis with and without cytokinesis, (2) in cells with an asymmetric architecture perpendicular to the direction of the mitotic spindle or with an asymmetry caused by the lateral location of an obstructing structure, and (3) in multinucleate cells that are too large to be cleaved by a circular furrow.

One point to be clarified in the context of unilateral furrowing is the role of myosin-II. Cytokinesis in *D. discoideum* is not exceptional, in the sense that myosin-II accumulates in the furrow region [16] and cytokinesis is impaired in myosin-II-null mutants [17,18,19]. However, there are two features that are remarkable. First, cytokinesis is strongly inhibited only if cells are cultivated in shaken suspension. When attached to an adhesive substrate surface, the mutant cells are capable of performing cytokinesis, albeit less efficiently than wild-type cells: this means they are forming a cleavage furrow linked to mitosis [20]. Second, in multinucleate myosin-II-null cells grown in shaken suspension, nuclei divide synchronously, but cytokinesis is impaired. When brought into contact with an adhesive substrate surface, these large cells form multiple cleavage furrows ingressing from their border [21]. 

A non-motor protein required for cytokinesis in *D. discoideum* is cortexillin that causes anti-parallel bundling of actin filaments [22]. Three isoforms, cortexillin I to III, form preferentially heterodimers [23,24]. Cells lacking both cortexillins I and II show severely impaired cytokinesis [22]. Cortexillin accumulates in the cleavage furrow [24,25,26] and has been proposed to interact there with myosin-II in a mechanosensory control system of contractility [27]. We have used GFP-cortexillin I to visualize cleavage furrows in myosin-II-null cells, where cortexillin accumulates at higher levels than in wild-type cells [28].

In order to study unilateral furrowing in cells other than myosin-II-null cells, we have produced multinucleate wild-type cells of *D. discoideum* through electric pulse-induced cell fusion [29]. We show that these fused cells are optimally suited to study cytokinesis by the ingression of unilateral furrows and compare them with multinucleate myosin-II-null cells. 

## 2. Materials and Methods

### 2.1. Cell Strains and Culture Conditions

Fluorescent proteins were expressed in the AX2-214 strain of *D. discoideum* [30] or in the HS2205 strain derived from it. In HS2205, myosin-II heavy chain has been deleted [19]. In the AX2-214 strain, GFP-myosin-II [31] together with mRFPM-α-tubulin [32], GFP-α-tubulin [21] together with mRFP1-histone 2B, or mRFPM-LimEΔ [32] together with GFP-α-tubulin were expressed. In the HS2205 strain, GFP-cortexillin I [28] was expressed together with mRFPM-histone 2B. 

### 2.2. Design of mRFP-Histone 2B Vectors

For the expression of histone 2B C-terminally of mRFP, two vectors were constructed, one conferring resistance to blasticidin, the other to hygromycin. For the blasticidin vector, the coding sequence of the *D. discoideum* histone variant H2Bv3 (DDB0231622|DDB_G0286509) was cloned into the *Eco*RI-site 3′ of mRFP1 [33] and expressed under control of an actin-15 promoter, using a pDEX-based vector conferring resistance to blasticidin [34].

To construct an expression vector with a hygromycin selection marker [35], the cassette A15P-mRFPmars-A8T consisting of actin-15 promoter, mRFPmars coding region [32], and actin-8 terminator was cloned between the *Sma*I and *Sph*I sites of the multiple cloning site of the pGEM7-based plasmid pHygTm(plus)/pG7 (a kind gift of Jeff Williams and Masashi Fukuzawa, University of Dundee). Subsequently, the coding sequence of histone H2Bv3 was inserted into the *Eco*RI site 3′ of mRFPmars.

### 2.3. Culture Conditions and Sample Preparation for Confocal Microscopy

Cells were cultivated in Petri dishes containing nutrient medium [36] supplemented with 10 µg/mL of blasticidin S (Gibco, Life Technologies Corporation, Grand Island, NY, USA), 10 µg/mL of geneticin (Sigma-Aldrich, St. Louis, MO, USA), or 33 µg/mL hygromycin B (EMD Millipore Corp., Billerica, MA, USA) at 21 ± 2 °C. 

For imaging, cells rinsed off the Petri dish were transferred to an HCl-cleaned cover-glass bottom dish (FluoroDish, WPI INC., Sarasota, FL, USA) and kept for 1 to 2 h in LoFlo medium (ForMedium Ltd., Norfolk, UK). The rate of mitosis could be increased by incubating the cells for about 20 h at 4 °C in Petri dishes with nutrient medium and subsequently bringing them to room temperature before transfer to LoFlo medium.

Large cells with wild-type AX2-214 background were produced by electric pulse-induced fusion as described by Gerisch et al. [29]. Myosin-II-null cells were cultivated in shaken suspension in nutrient medium for about 36 h to get large multinucleate cells [20]. Multinucleate cells were transferred onto HCl-cleaned cover-glass bottom dishes and incubated in LoFlo medium for about 1 h before imaging was started. Where indicated in the figure legends, cells were overlaid with a thin agarose sheet [37,38]. The velocity of unilateral furrow propagation was measured beginning at 1 µm of ingression.

### 2.4. Confocal Image Acquisition and Data Processing

For confocal images, a Zeiss LSM 780 microscope equipped with a Plan-Apo 63x/NA 1.46 oil immersion objective was used (Zeiss AG, Oberkochen, Germany). Images were processed using the image-processing package Fiji (http://Fiji.sc/Fiji) developed by Schindelin et al. [39] on the basis of ImageJ (http://imagej.nih.gov/ij). For bleach correction of the red channel, the total mean grey level for each image of a series was measured to calculate the percentage of bleaching, and accordingly, the brightness of the red channel was linearly enhanced.

For 3D rendering and animation, the images were first deconvolved with the software of Huygens Essential, version 18.04 (Scientific Volume Imaging b.v., Hilversum, The Netherlands) and then animated and displayed in UCSF Chimera, version 1.14 (https://www.cgl.ucsf.edu/chimera) [40].

## 3. Results

### 3.1. Mitosis in Wild-Type and Myosin-II-Null Cells

**Figure 1:**
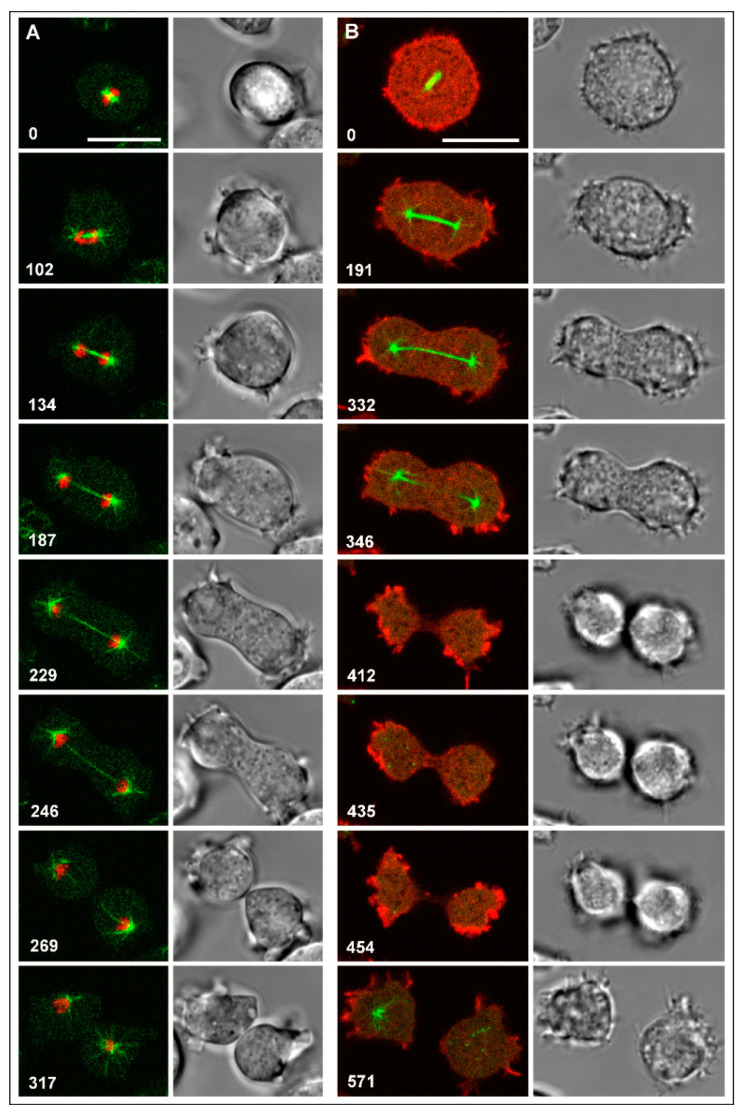
Mitosis and cytokinesis in wild-type cells of *Dictyostelium discoideum*. The left panels in the time series of (**A**) and (**B**) show confocal dual-color fluorescence images, the right panels show DIC bright-field images. Time after the first frame of each series is indicated in seconds. Scale bars, 10 µm. (**A**) A cell expressing GFP-α-tubulin as a label for the mitotic apparatus (green) and mRFP-histone 2B to visualize the chromosomes (red). The elongated spindle is disrupted between the 246 s and 269 s frames. The 317 s frame shows in the right cell radial microtubules connecting the centrosome with the cell cortex. (**B**) A cell expressing GFP-α-tubulin (green) and mRFP-LimEΔ as a label for filamentous actin (red). Fluorescence images are primarily focused on the spindle or on polar protrusions; for the 435 s frame, the focus was changed to the cleavage furrow, where little actin had accumulated. The cell was flattened by agarose overlay. The same sequence is shown in Supplementary Video S1. For a 3D display showing polar protrusions attached to the substrate surface, see Supplementary Video S2.

As a reference for mitosis, we will first show spindle dynamics and chromosome segregation in a wild-type cell containing a single dividing nucleus (Figure 1A and Supplementary Video S1). This cell expressed GFP-α-tubulin (green) as a constituent of the mitotic apparatus together with mRFP-histone 2B to label chromosomes (red). During metaphase (0-s frame), the two centrosomes stayed at a distance of only 1 to 2 µm from each other. Accordingly, the connecting spindle was short; subsequently it elongated, before disrupting in the middle. At anaphase, the two sets of daughter chromosomes immediately followed the separating centrosomes, which means that centromere-associated microtubules remained short. During the entire process, aster microtubules connected the centrosomes with the polar regions of the cell cortex. This example is representative of the dynamics of the mitotic apparatus in mononucleate and multinucleate wild-type or myosin-II-null cells (Table 1). Not seen in Figure 1 is the nuclear membrane, which separates the centrosomes from the intranuclear spindle in the semi-closed mitosis of *Dictyostelium* [41,42].

### 3.2. Cytokinesis in Mononucleate Wild-Type and Myosin-II-Null Cells

Upon disruption of the spindle, the cleavage furrow ingressed in the midplane of wild-type cells, as shown in Figure 1A. The furrow separated the daughter cells except for a thin tubular bridge that was finally disrupted by a dynamin A-dependent mechanism [43]. Filamentous actin accumulated most prominently in rounded protrusions at the polar regions of the dividing cell and only faintly in the cleavage furrow (Figure 1B, Supplementary Video S1 and Supplementary Video S2).

**Figure 2:**
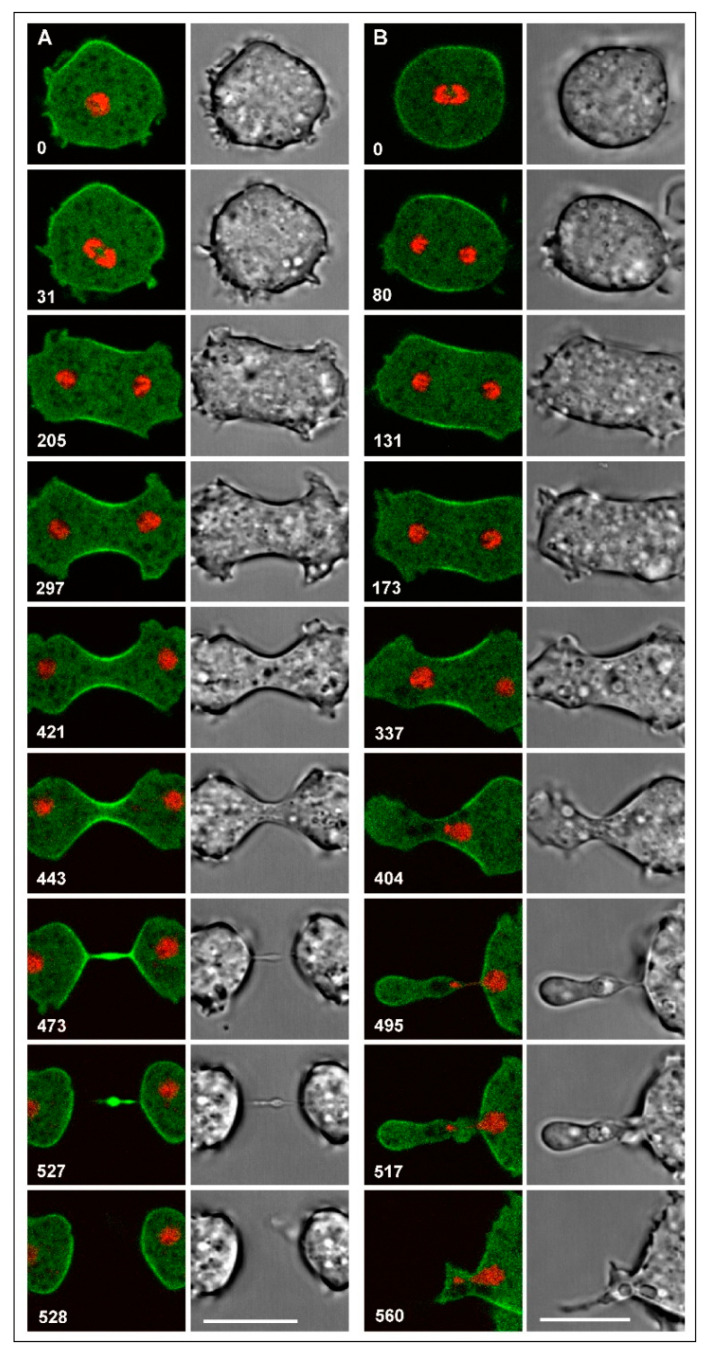
Successful and failing cytokinesis in mononucleate myosin-II-null cells. Cells attached to a glass surface are shown in confocal fluorescence (left panels) and bright field images (right panels). The cells expressed GFP-cortexillin I (green), together with mRFP-histone 2B as a label of the chromosomes. Time is indicated in seconds after the first frame. Scale bars, 10 µm. The entire sequences of (**A**) and (**B**) are shown in Supplementary Video S3. (**A**) Successful cell division showing cortexillin accumulating in the cleavage furrow that divides the cell between the two daughter nuclei. (**B**) Unsuccessful cytokinesis, which begins like the successful one with the accumulation of cortexillin in the midzone. However, subsequently, the furrow becomes asymmetric, and the left nucleus slips toward the right, the now anucleate part of the cell becoming integrated into the binucleate one. This cell was gently compressed by an agar overlay, so that the upper and lower surfaces were brought into parallel planes, and the dividing nuclei were kept in focus.

Mononucleate myosin-II-null cells that completed cytokinesis, accumulated cortexillin in the cleavage furrow (Figure 2A and Supplementary Video S3, top). Instability of furrow positioning was indicated by those cells in which the furrow, initiated as usual in the middle of the cell, slipped to one side, such that it would separate a binucleate portion from an anucleate one (Figure 2B and Supplementary Video S3, bottom). In that way, the furrow became located on top of aster microtubules, which did not support its further ingression. The anucleate portion was rather retracted, resulting in failure of cytokinesis.

### 3.3. Unilateral Cleavage Furrows in Multinucleate Wild-Type Cells

In all kinds of the multinucleate cells tested, that is, in fused wild-type cells, myosin-II-null cells pre-grown in suspension, and cortexillin I- and II-null cells, the nuclei divide synchronously. To label unilateral cleavage furrows in multinucleate cells, we alternatively used two proteins that are involved in cleavage furrow formation accompanying mitosis, i.e., the two-headed motor protein myosin-II [31] and cortexillin, an anti-parallel bundler of actin filaments [22].

**Figure 3:**
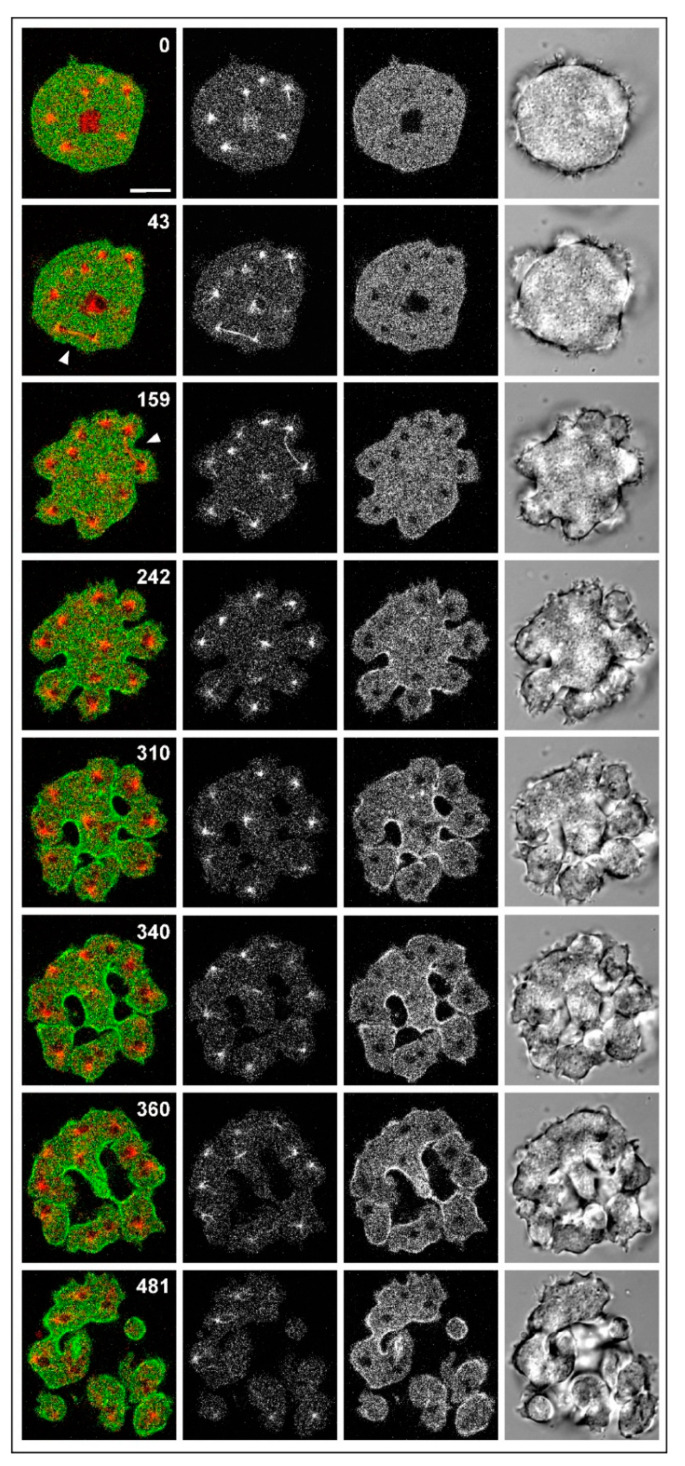
Unilateral furrows in a multinucleate wild-type cell produced by electric pulse-induced fusion. The cells fused expressed mRFP-α-tubulin (red) and GFP-myosin-II heavy chains (green in the merged panels). Confocal fluorescence images show the labels merged (left) but also separated (second row: α-tubulin; third row: myosin-II). On the right, DIC bright-field images are shown. In the merged panels, a straight spindle before furrow ingression and a spindle bent after the onset of furrowing are indicated by arrowheads. In the middle of the large cell, an interphase cell was apparently entrapped by accident (seen in the 0 and 43 s frames). Time is indicated in seconds after the first frame. Scale bar, 10 µm. The entire time series from which these images were taken is covered in Supplementary Video S4.

The division of large wild-type cells produced by electric pulse-induced fusion is exemplified in Figure 3 and Supplementary Video S4, showing a cell that expressed mRFP-α-tubulin to label the mitotic apparatus together with GFP-myosin-II heavy chains to visualize the accumulation of myosin in unilateral furrows. The mitotic apparatus showed the docking of aster microtubules to the cell cortex, as previously observed in myosin-II-null cells [20]. Docking resulted in bending of the spindle (159 s frame) and in the induction of protrusions where furrow formation was inhibited. Again, in accord with previous findings on myosin-II-null cells [21], furrows ingressed at spaces not occupied by microtubule asters, independent of whether or not these spaces were bridged by a spindle.

**Figure 4:**
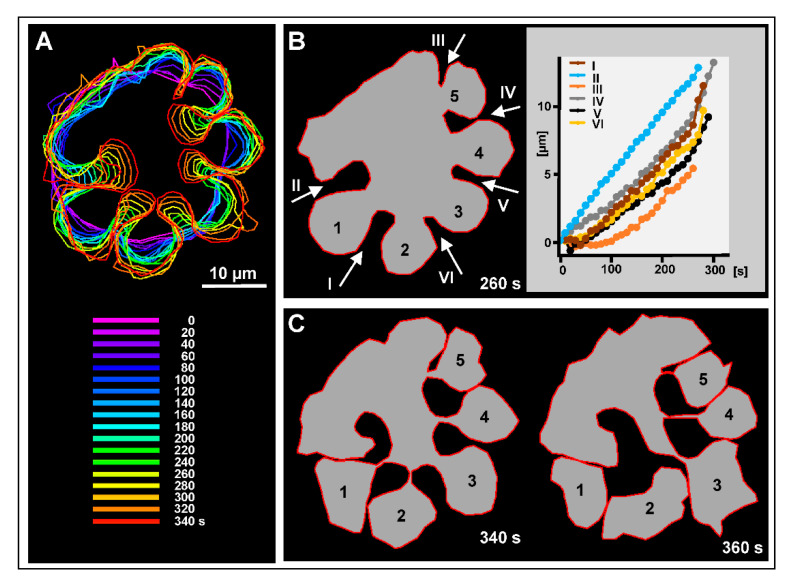
Progression of cleavage furrows in the multinucleate wild-type cell of Figure 3. (**A**) Color-coded cell boundaries within the 0 to 340 s time span derived from the confocal fluorescence images of myosin-II. (**B**) Left panel: cell boundary at the 260 s time point, showing unilateral cleavage furrows I to VI and incipient daughter cells 1 to 5. Right panel: progression of the six cleavage furrows, measured in the direction indicated by the arrows in the left panel. (**C**) Comparison of cell boundaries at the 340 s and 360 s time points. Within the time span of 20 s, the daughter cells 1 and 2 became disconnected, while separation of the incipient daughter cells 3 to 5 proceeded. In all these cases, abscission occurred obliquely to the initial furrowing. The drawings in (**B**) and (**C**) show the cell shapes in the confocal plane, with additional information on connectivity gathered from the bright-field images.

The unilateral furrows in wild-type cells ingressed with an average velocity of 1.7 µm × min^−1^, with no appreciable decrease of the velocity in the course of ingression (Figure 4A,B). They widened during ingression and tended to cooperate with neighboring furrows to form constricting rings that separated mono- or oligo-nucleate portions from the multinucleate cell mass (Figure 4C). These cases are of interest because here the final constriction was not a continuation of the initial furrow ingression but proceeded laterally between two furrows.

### 3.4. Division of Multinucleate Myosin-II-Null Cells

**Figure 5:**
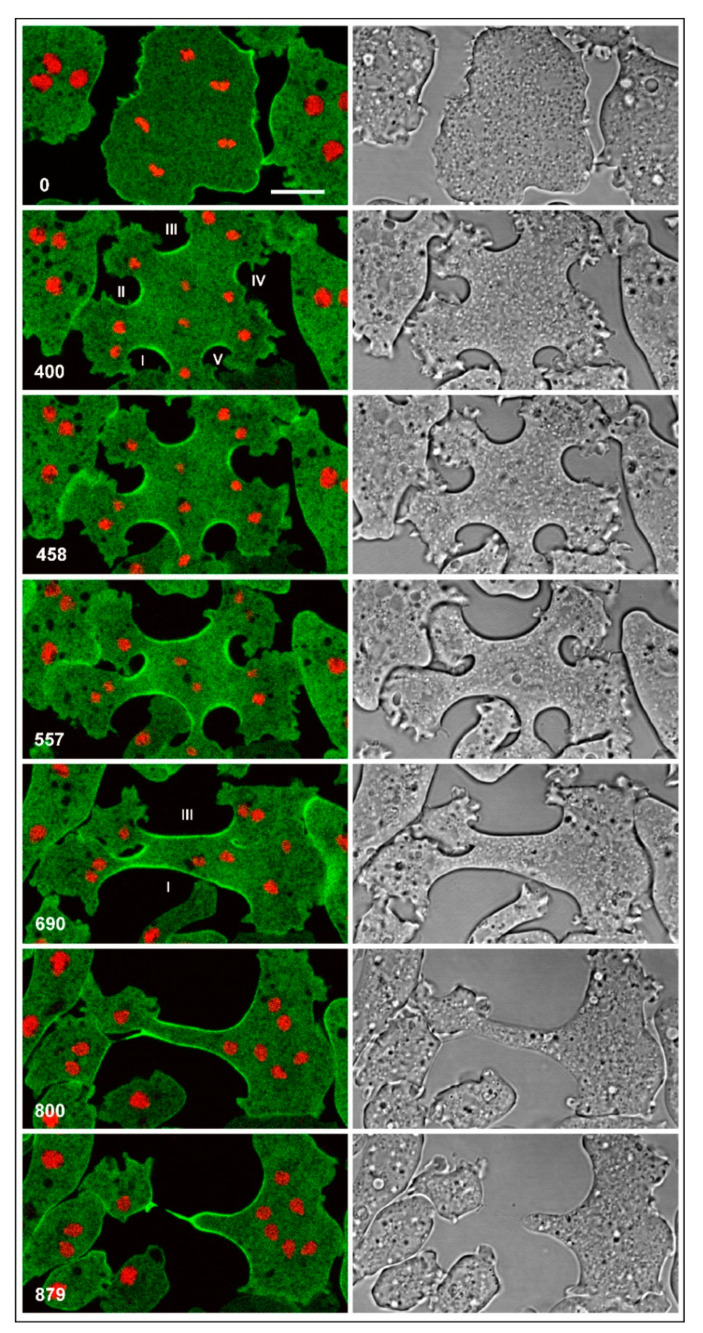
Synchronous mitosis and division of a multinucleate myosin-II-null cell. Like the cells in Figure 2, this cell expressed GFP-cortexillin I (green) and mRFP-histone 2B (red). Initially, five unilateral furrows formed that were enriched in cortexillin (400 s to 557 s frames). Later on, two long furrows prevailed (690 s and 800 s frames), and a final abscission occurred between them (879 s frame). The cell was flattened by agarose overlay. Time is indicated in seconds after the first frame. Scale bar, 10 µm. The entire sequence is shown in Supplementary Video S5.

**Figure 6:**
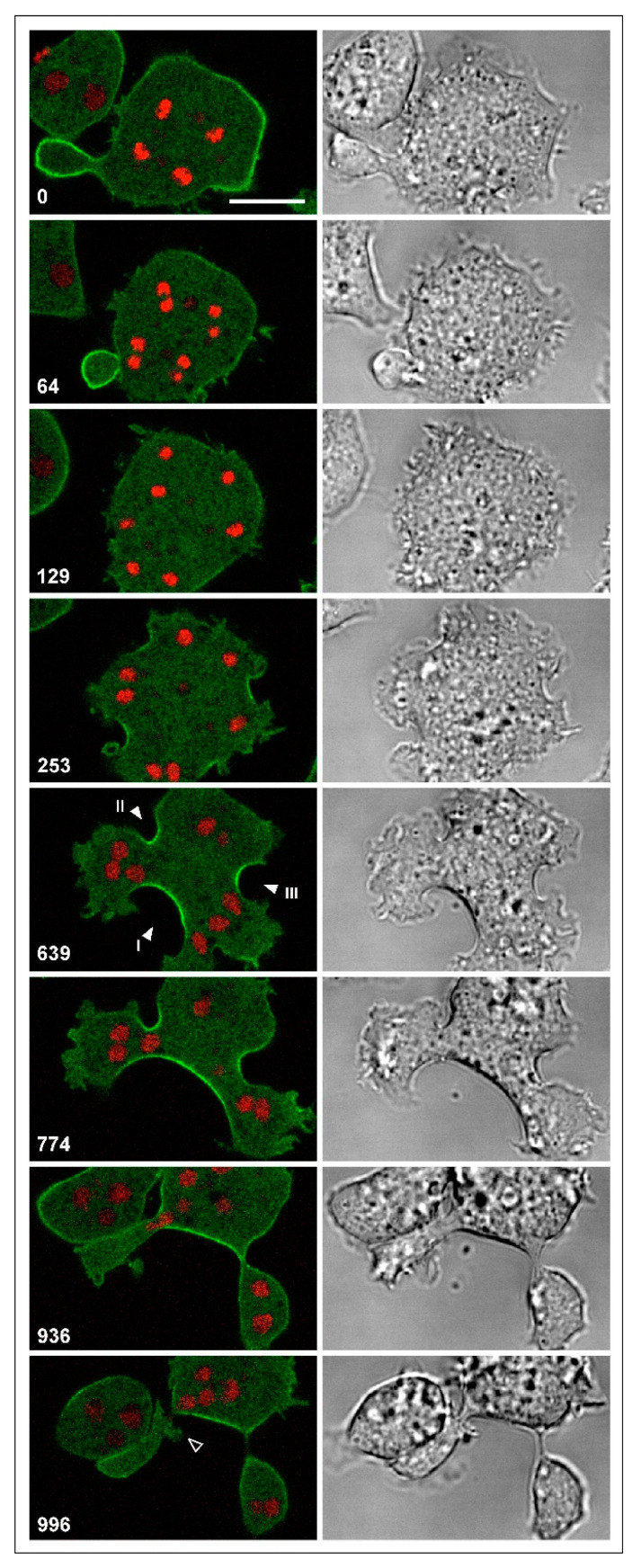
A multinucleate myosin-II-null cell undergoing successful and unavailing cytokinesis. The cell is labeled, like the cell in Figure 5, for cortexillin I (green) and for the chromosomes (red). Three unilateral furrows are indicated by arrowheads in the 639 s frame. Between furrow I and III, abscission of a daughter cell proceeds, whereas between furrows I and II three nuclei slip from the left portion into the major cell body (774 s and 936 s frames). Consequently, the anucleate part is united with the major cell body, forming protrusions toward the latter (open arrowhead in the 996 s frame). Time is indicated in seconds after the first frame. Scale bar, 10 µm. The same sequence is shown in Supplementary Video S6.

Multinucleate myosin-II-null cells can divide by unilateral furrows to which cortexillin is localized. However, these mutant cells appeared to inefficiently restrict the expansion of these furrows: while some furrows strongly expanded before the daughter cells were separated, other furrows stopped growing. An extreme example is shown in Figure 5 and Supplementary Video S5, with two major and three minor furrows. The multinucleate cell shown in Figure 6 and Supplementary Video S6 also formed a long furrow, the only one which continued to propagate (indicated as furrow 1 by the arrowhead). In cooperation with furrow 3, the broad furrow 1 gave rise to a daughter cell, while interaction with furrow 2 failed. All three nuclei in the incipient daughter cell slipped through the gap into the major part of the cell (774 s and 936 s frames). The anucleate remnant directed protrusions toward the major part (996 s frame) and reintegrated, similar to the anucleate portion of the mitotic cell in Figure 2B. 

**Figure 7:**
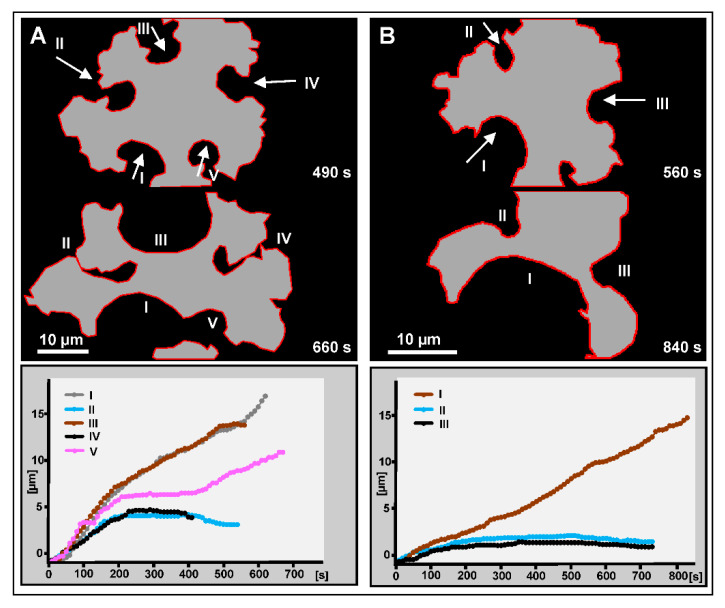
Progression and arrest of unilateral furrows in the myosin-II-null cells shown in the time series of Figure 5 and Figure 6. (**A**) The cell shown in Figure 5 and in Supplementary Video S5 forms two progressing furrows (I and III) and two furrows that stop growing (II and IV). Furrow V enters a phase of arrest before it merges with the broad furrow I and continues propagating as part of a joint furrow. (**B**) The cell shown in Figure 6, with furrow I continuously progressing, and furrows II and III coming to a halt. The top and middle panels show the cell boundaries at earlier and later stages of furrow incision. The indicated times correspond to the time scales in Figure 5 and Figure 6, respectively. Zero-time in the curves is set to the beginning of furrow ingression.

Figure 7 provides quantitative data on furrow progression and arrest in the cells shown in Figure 5 and Figure 6. Figure 7A refers to the cell in Figure 5, in which furrows 2 and 4 stopped growing, while furrows 1 and 3 progressed. Furrow 5 showed a hybrid behavior: it first stopped and subsequently united with furrow 1 and resumed growing. Figure 7B, which refers to the cell in Figure 6, shows a clear distinction between furrow I that progressed continuously and furrows II and III that completely stopped after an initial phase of incision. These data indicate that in the absence of myosin-II, unilateral furrows can progress with almost the same velocity as in wild-type background and that two furrows can join to separate daughter cells. However, often furrows come to an early arrest, and the separation of daughter cells may fail.

A summary of the data obtained for myosin-II-null cells is provided in Table 2, showing that altogether, half of the mutant cells succeeded in completing cytokinesis when attached to an adhesive substrate surface. We wish to add that the high proportion of failures held only for myosin-II-null cells grown axenically in nutrient medium. Cells fed with bacteria performed cytokinesis with higher efficiency, as previously reported [28].

## 4. Discussion

In *D. discoideum*, myosin-II-null as well as wild-type cells provide the possibility of studying the division of mononucleate and multinucleate cells and thus of normal and unilateral cleavage furrow formation in an identical genetic background. The inability of myosin-II-null cells to perform cytokinesis in suspension and the support of their mitotic division by an adhesive substrate made it easy to compare mononucleate and multinucleate cells in the absence of myosin-II. 

Both wild-type and myosin-II-null cells showed how two unilateral furrows join to form a ring that separates a daughter cell from the multinucleate cell body, but the fused wild-type cells were superior because of the higher efficiency of furrow formation (Figure 3). In myosin-II-null cells, this ring constricted by a myosin-II independent mechanism, though frequently failed to complete cytokinesis (Figure 2). Together, the data obtained in mononucleate and multinucleate myosin-II-null cells are in accord with previous findings indicating that myosin-II stabilizes the position of the cleavage furrow [26,44], preventing its lateral sliding (Figure 2B) or expansion (Figure 5 and Figure 6).

The sites of unilateral cleavage furrows are negatively controlled by positioning of the microtubule asters that emanate from the centrosomes during mitosis. The aster microtubules induce the cell cortex to ruffle, thus preventing the formation of a furrow. The cells are cleaved at spaces between asters, even if the flanking asters are not connected by a spindle [21]. The ingression of cleavage furrows between centrosomes that are not connected by a spindle relates cytokinesis in multinucleate *Dictyostelium* cells to furrow formation in sand dollar eggs [45]. However, the argument that these furrows are induced by aster microtubules [46] is probably not applicable to *Dictyostelium*. The asters may only act in inducing actin-rich protrusions, which correspond to polar regions in mononucleate cells ([20,21] and Figure 1B of the present paper). In multinucleate cells, there is no recognizable microtubule structure that may act as a source of signals for furrow ingression in the spindle-free inter-centrosomal spaces.

A protein important for the formation of cleavage furrows in *Dictyostelium* is cortexillin, which bundles actin-filaments [22]. Cortexillin may serve a function similar to that of anillin, another actin-bundling protein [47,48], which is missing in *Dictyostelium*. In various other cells, anillin is recruited to the furrow region [49] to fulfil multiple functions in cytokinesis [50]. In vitro, anillin can be shown to act independently of myosin in the constriction of an actin ring [51].

Cytokinesis in *Dictyostelium* cells is extremely adaptable since, depending on environmental conditions, different mechanisms are exploited on the route to complete the separation of the daughter cells [52]. The unilateral furrows studied here indicate that for initiation of a cleavage furrow, no contractile ring is required. The accumulation of myosin-II and cortexillin in unilateral furrows formed in multinucleate wild-type cells identifies two molecular players in the ingression of these furrows, which are established constituents of the machinery involved in constricting the ring-shaped furrows in mononucleate cells.

## 5. Conclusions

**Figure 8:**
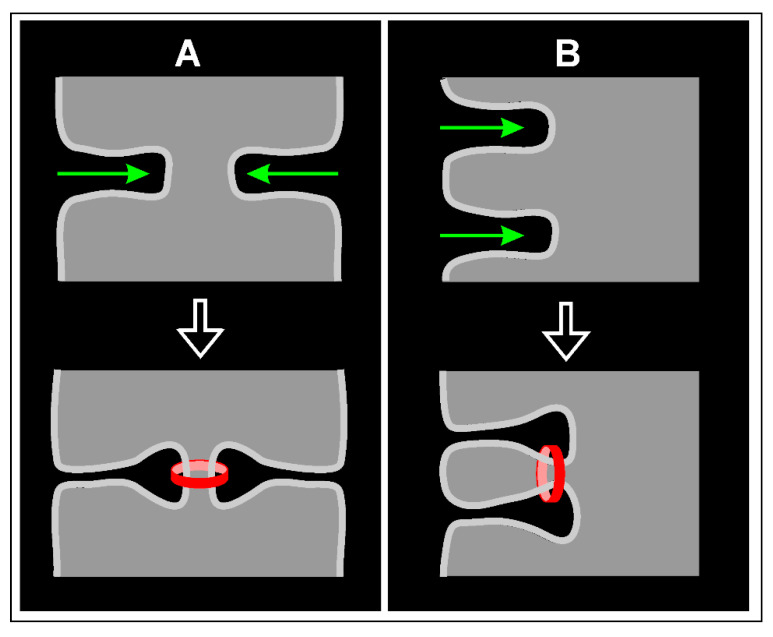
Diagram of cytokinesis in multinucleate cells, illustrating the variable spatial relationship of initial furrow ingression (green arrows) and final abscission (red ring). (**A**) Two furrows ingressing from opposite sides, and separation proceeding along the furrow directions. (**B**) Two furrows ingressing in parallel directions, and abscission occurring obliquely to these directions.

A peculiar feature of cytokinesis in multinucleate cells of *Dictyostelium* is the variable geometric relationship between furrow ingression and the subsequent ring contraction that finally results in the abscission of daughter cells (Figure 3). Ring formation may just be a continuation of the initial ingression events. This is the case when two furrows meet from opposite sides (Figure 8A). However, different from cytokinesis in mononucleate cells, the rings may also be formed laterally with respect of the initial ingression (Figure 8B), thus separating the entire process of cleavage into two distinct phases.

## Figures and Tables

**Table 1:** Mitotic spindles in wild-type and myosin-II-null cells.

Mononucleate Cells	Wild-Type	Myosin-II-Null
Velocity of spindle elongation	44 ± 15 s.d. nm/s	56 nm/s
Maximal spindle length	11.8 ± 1.3 s.d. µm	14.0 µm
Number of measured cells	7	1 for velocity, 3 for spindle length
**Multinucleate Cells**	**Wild-Type**	**Myosin-II-Null**
Velocity of spindle elongation	46 ± 11 s.d. nm/s	51 ± 14 s.d. nm/s
Maximal spindle length	14.9 ± 2.1 s.d. µm	13.9 ± 1.4 s.d. µm
Number of measured spindles	17 in 5 cells	18 in 6 cells

The velocity of elongation was measured between 3 and 10 µm of spindle length. The spindle length between the center of the centrosomes was determined in the frame before the spindle was disrupted.

**Table 2:** Mitosis in myosin-II-null cells attached to a glass surface with successful or unsuccessful cytokinesis.

Pre-Culture	Dividing Nuclei	Completed	Failed
(1) On substrate	Mono-nucleate	3	4 [1]
	Multi-nucleate	2 [1]	1 [1]
(2) Shaken culture	Mono-nucleate	2	1
	Multi-nucleate	20	9
(3) Electric pulse-fused	Mono-nucleate	2	0
	Multi-nucleate	8	3
Total		37	18

The cells were pre-cultured under one of three different conditions: (1) in a plastic Petri dish; (2) in shaken suspension; (3) in Petri dishes and subsequently fused. With three exceptions, cell division was observed without application of an agar overlay. The number of cells which were gently compressed by an overlay is provided in squared brackets.

## Supplementary Materials

The following are available online at https://www.mdpi.com/2073-4409/9/6/1493/s1, Video S1: Mitosis and cytokinesis in a wild-type cell of *Dictyostelium discoideum*, Video S2: Animation of three stages of cell division of a 3D-rendered wild-type cell of *D. discoideum*, Video S3: Successful (top) and failing (bottom) cytokinesis in myosin-II-null cells, Video S4: A multinucleate wild-type cell produced by electric pulse-induced fusion, Video S5: Synchronous mitosis and division of a multinucleate myosin-II-null cell, Video S6: A multinucleate myosin-II-null cell.
