# Supplementary material for: Unilateral Cleavage Furrows in Multinucleate Cells
Source: Cells. 2020 Jun 18;9(6):1493. doi: 10.3390/cells9061493 (PMC7349700; doi:10.3390/cells9061493)
Supplement: Supplementary file 1 [file cells-09-01493-s001.zip › cells-739708_Bindletal_Video Legends_rev.pdf]

Videos are published on Zenodo: https://zenodo.org/record/3859782
DOI: 10.5281/zenodo.3859782

Supplementary Materials

Supplementary Video Legends

Video 1. Mitosis and cytokinesis in a wild-type cell of *Dictyostelium discoideum*.
The cell expressed GFP-α-tubulin (green) together with mRFP-LimEΔ as an actin label (red). The left panel shows confocal dual-color fluorescence images, the right panels DIC bright-field images.
The cell has been slightly flattened by agarose overlay. Focus has been changed at the 417-s to 420-s frames. The 5-s frame in the video corresponds to the 0-s frame in Figure 1B. Frame-to-frame interval is 1 s. Bar, 10 µm.

Video 2. Animation of three stages of cell division of a 3D rendered wild-type cell of *D. discoideum*. The cell expressed GFP-α-tubulin (green) together with mRFP-LimEΔ as an actin label (red). Time is indicated in seconds. Bar, 10 µm.

Video 3. Successful (top) and failing (bottom) cytokinesis in myosin-II-null cells. Mitoses of mononucleate cells on a glass surface are shown. The cells expressed GFP-cortexillin I (green), together with mRFP-histone 2B as a label of the chromosomes. The left panels show confocal dual-color fluorescence images, the right panels DIC bright-field images. In the upper panels the 1-s frame in the video corresponds to the 0-s frame in Figure 2A. In the lower panels the 57-s frame in the video corresponds to the 0-s frame in Figure 2B. The cell on bottom has been slightly flattened by agarose overlay. Frame-to-frame interval is 1 s. Bar, 10 µm.

Video 4. A multinucleate wild-type cell produced by electric-pulse induced fusion. The cells fused expressed mRFP-α-tubulin (red) and GFP-myosin-II heavy chains (green). The left panel shows confocal dual-color fluorescence images, the right panel DIC bright-field images. The 19-s frame in the video corresponds to the 0-s frame in Figure 3. At the beginning of the sequence, an interphase cell is seen to be surrounded by the syncytium. In the 49-s to 180-s frames, spindles are bent when the aster microtubules interact with the cell cortex. Frame-to-frame interval is 1 s. Bar, 10 µm.

Video 5. Synchronous mitosis and division of a multinucleate myosin-II-null cell. This cell expressed GFP-cortexillin I (green) and mRFP-histone 2B (red). The left panel shows confocal dual-color fluorescence images, the right panel DIC bright-field images. At the 446-s and 597-s frames the focus has been on the bottom surface of the cell. The 60-s frame in the video corresponds to the 0-s frame in Figure 5. The cell has been flattened by agarose overlay. Frame-to-frame interval is 1 s. Bar, 10 µm.

Video 6. A multinucleate myosin-II-null cell. The cell expressed GFP-cortexillin I (green), together with mRFP-histone 2B as a label of the chromosomes. The left panel shows confocal dual-color fluorescence images, the right panel DIC bright-field images. Division on the left side fails, and three nuclei slip through the furrow toward the right. The 9-s frame in the video corresponds to the 129-s frame in Figure 6. Frame-to-frame interval is 1 s. Bar, 10 µm.
